# A Mendelian randomization approach to study the causal association between four types of endometriosis and immune cells: experimental studies

**DOI:** 10.1097/JS9.0000000000001909

**Published:** 2024-07-03

**Authors:** Wangshu Li, Xiuying Wang, Xu Zhang, Shu Sun, Jiuxiang Feng, Aziz ur Rehman Aziz

**Affiliations:** a Key Laboratory for Early Diagnosis and Biotherapy of Malignant Tumors in Children and Women, Dalian Women and Children’s Medical Group, Dalian; b Department of Obstetrics and Gynecology, Shengjing Hospital of China Medical University, Shenyang, Liaoning; c The Second Affiliated Hospital of Harbin Medical University, Harbin Medical University, Harbin, Heilongjiang, People’s Republic of China

Highlights

- This study uses a two-sample Mendelian randomization method to uniquely establish causal relationships between immune cells and four different subtypes of endometriosis, offering a novel perspective on the immunological underpinnings of the disease.
- It includes thorough sensitivity analyses to ensure the reliability of the findings, effectively addressing inconsistencies found in prior research.
- The study identifies specific immune cells involved in different types of endometrioses, providing a genetic basis for targeted therapeutic strategies.

Endometriosis (EM) is a condition where endometrial tissue grows outside the uterus, causing chronic inflammation. It affects about 10% of women of childbearing age, impacting reproductive health and quality of life. EM is classified into four types based on lesion location: peritoneal (PE), ovarian (OE), deep infiltrating (DIE), and other locations such as intestinal (IE). Each type varies in clinical features, causes, and prognosis. Inflammation due to immune system dysregulation is a key factor in EM, leading to cell proliferation and infiltration^1,2^. Various immune cells contribute to the formation and development of endometrial lesions in EM. However, their exact role is debated due to conflicting findings, especially regarding natural killer (NK) cell levels. Research is hindered by blood contamination in samples and confounding factors like age, environment, diet, and lifestyle, resulting in inconsistent results and limiting further investigation into the connection between immune cells and EM^3,4^.

The study investigates the causal relationship between immune cells and four types of EM using genome-wide association study (GWAS) data by conducting a two-sample Mendelian randomization (MR) analysis (Fig. 1). MR employed genetic variations as instrumental variables (IVs) to represent risk factors, ensuring they meet three key assumptions for valid causal inference^5^. The GWAS data included 731 immunophenotypes, categorized into absolute cell counts, median fluorescence intensities, morphological parameters, and relative cell counts, analyzed from 3757 European individuals. Around 22 million single-nucleotide polymorphisms (SNPs) were examined using high-density arrays and sequence-based imputation, with adjustments for covariates such as sex and age to enhance statistical rigor. EM data were sourced from the FinnGen Consortium (https://www.finngen.fi/fi), analyzing genomic and health data from 500 000 Finnish participants as of January 2022 (Supplementary Table S1, Supplemental Digital Content 1, http://links.lww.com/JS9/D10). PLINK software was used for IV selection and clumping procedures, to calculate the proportion of phenotypic variation explained, and to use the F-statistic to prevent weak IV bias. A total of 108 robust IVs were retained for each type of EM after discarding those with low F-statistics to ensure analysis reliability. R 3.6.2 software was used to assess relationships using various MR techniques, including Inverse Variance Weighting (IVW), weighted median estimator (WME), MR-Egger regression, simple mode, and weighted mode, executed with the ‘Mendelian-Randomization’ package (version 0.4.2). Cochran’s *Q* statistic was used to detect heterogeneity, with the random effects IVW method applied if heterogeneity was found. MR-Egger was employed to detect horizontal pleiotropy, ensuring the robustness of the analysis.

**Figure 1:**
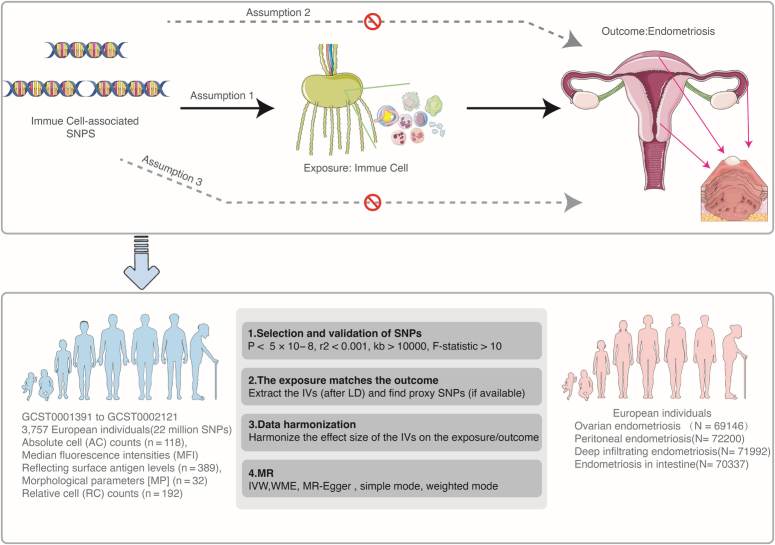
Framework for evaluating the causal impact of immune cell traits on endometriosis (EM) subtypes. IVs, instrumental variables; IVW, inverse variance weighting; LD, linkage disequilibrium; MR, Mendelian randomization; SNPs, single-nucleotide polymorphisms; WME, weighted median estimator.

We identified several immune phenotypes associated with PE, OE, DIE, and IE through data analysis (Fig. 2 and Supplementary Table S2, Supplemental Digital Content 2, http://links.lww.com/JS9/D11). Among the various immune cells involved in the pathogenesis of OE, regulatory T cells (Tregs), monocytes, myeloid cells, and conventional dendritic cells are predominant. The IVW method identified that CD3 and CD39 subtypes are primarily implicated in the OE occurrence. For monocytes, the CD40 subtype has demonstrated a protective effect against OE.

**Figure 2:**
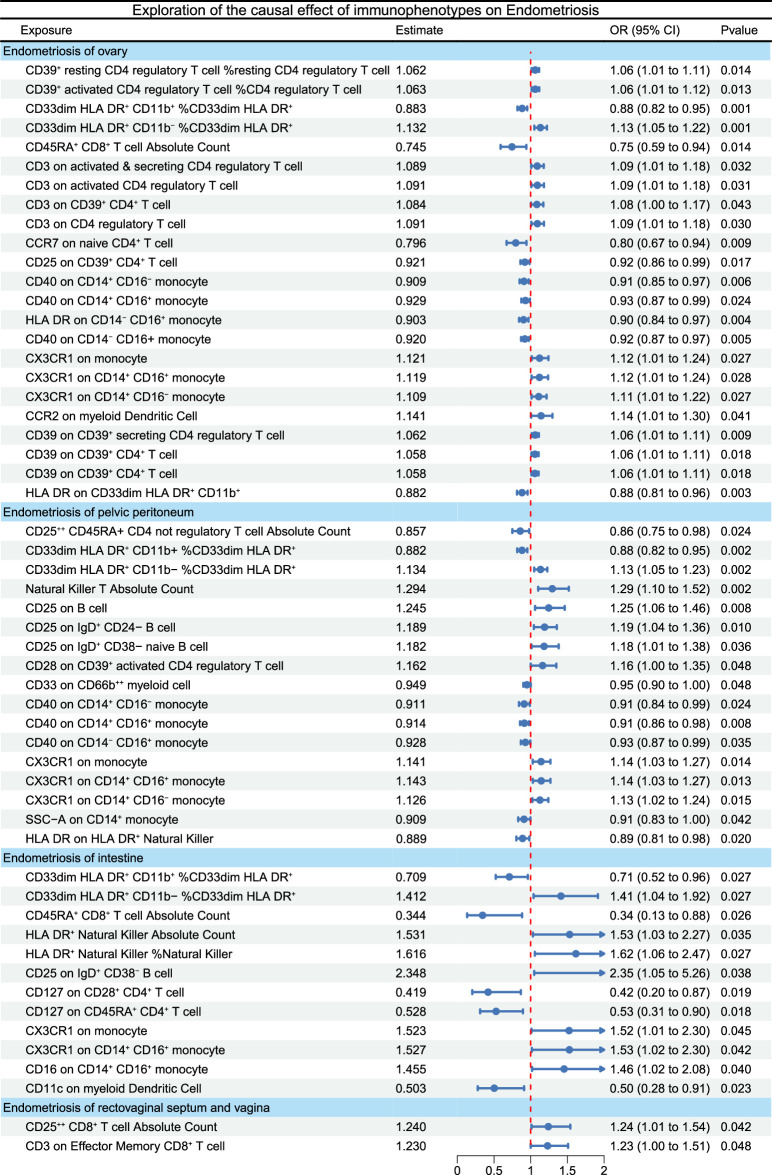
Forest plot depicting the association between immunophenotypes and endometriosis (EM) subtypes by using inverse variance weighting (IVW) methods.

We observed associations between peritoneal EM and CD25 expression on B cells, CD25 on IgD^+^ CD24^−^ B cells, and CD25 on IgD^+^ CD38^−^ naive B cells. The absolute count of CD25^++^ CD45RA^+^ CD4 non-regulatory T cells and CD33^dim^ HLA DR^+^ CD11b^+^%CD33^dim^ HLA DR^+^ myeloid cells showed a negative association and CD28 expression on CD39^+^ activated CD4 Tregs and CD33^dim^ HLA DR+ CD11b^−^ %CD33^dim^ HLA DR^+^ myeloid cells were positively associated with PE. Similarly, associations between PE and the absolute count of T cells and HLA DR expression on HLA DR^+^ NK cells were observed.

Compared to other subtypes, there are fewer immune cell types associated with DIE. Only the absolute count of CD25^++^ CD8^+^ Tregs and CD3 on Effector Memory CD8^+^ T cells have a positive causal relationship with DIE. The CD25 on IgD^+^ CD38^−^ B cells, CD33^dim^ HLA DR^+^ CD11b^−^ %CD33^dim^ HLA DR^+^, CD16 on CD14^+^ CD16^+^ monocytes, CX3CR1 on CD14^+^ CD16^+^ monocytes, and CX3CR1 on monocytes, HLA DR^+^ NK % NK and HLA DR^+^ NK absolute count have a positive causal relationship with IE. In comparison, the CD127 on CD28^+^ CD4^+^ T cells, CD127 on CD45RA^+^ CD4^+^ T cells, and CD33^dim^ HLA DR^+^ CD11b^+^ %CD33^dim^ HLA DR^+^ have a negative causal relationship with IE.

CD33^dim^ HLA DR^+^ CD11b^+^ %CD33^dim^ HLA DR^+^, CD33^dim^ HLA DR^+^ CD11b^−^ %CD33^dim^ HLA DR^+^, CX3CR1 on monocytes, and CX3CR1 on CD14^+^ CD16^+^ monocytes all have significant causal relationships with different types of EM. Among them, CD33^dim^ HLA DR^+^ CD11b^+^ %CD33^dim^ HLA DR^+^ mainly shows a negative causal relationship, while the other three types of immune cells all show positive causal relationships. Moreover, we identified unique characteristic immune cells of each subtype (Fig. 3). The detailed information from the sensitivity analysis of all results has validated the robustness of the observed causal associations (Supplementary Table S3, Supplemental Digital Content 3, http://links.lww.com/JS9/D12).

**Figure 3:**
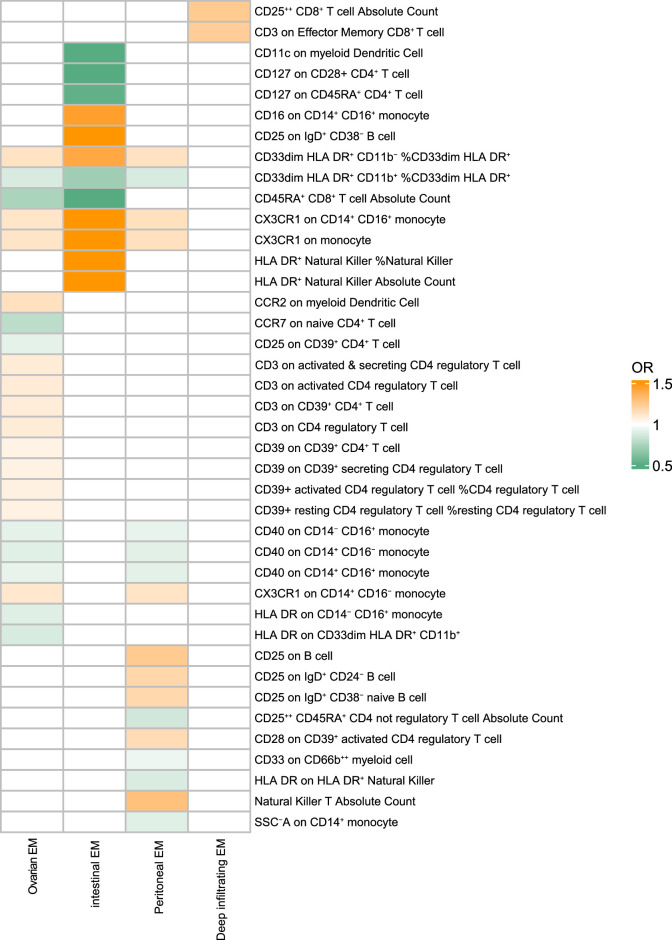
Heatmap of immunophenotype associations with endometriosis (EM) subtypes.

Our findings reveal a wider variety of associated immune cell types for OE, PE, and IE, while the pathogenesis of DIE may be inconsistent with other types. This aligns with the conclusions drawn in previous studies^6–10^ and validates the hypothesis that different EM subtypes possess distinct pathological mechanisms and clinical manifestations. Our study primarily includes data from European populations, which covers a wide range of immune cells but is not exhaustive. We used a few GWAS databases to study different subtypes of EM. The DIE data mainly comes from the vaginal fornix and rectovaginal septum, which may not fully represent this subtype. Additionally, our focus on intestinal cases for other EM locations may not be as relevant for rarer forms of EM. Despite these limitations, we confirmed our findings where possible. Future research should include more anatomical locations and clinical variants for a comprehensive understanding of EM’s genetic and molecular mechanisms.

## Ethical approval

All the data are publicly available and no ethical approval or consent for publication was required.

## Consent

Not applicable.

## Source of funding

This study was supported by the Dalian City Outstanding Young Science and Technology Talent Program, No. 2023RY020.

## Author contribution

W.L.: data curation, funding acquisition, investigation, methodology, and writing – original draft; X.W.: conceptualization, resources, and writing – review and editing; X.Z.: data curation, methodology, and writing – original draft; S.S.: writing – original draft; J.F.: conceptualization, project administration, and writing – review and editing; A.R.A.: conceptualization, data curation, supervision, and writing – review and editing. All authors have read and approved the final manuscript for publication.

## Conflicts of interest disclosure

The authors declare that they have no conflicts of interest.

## Research registration unique identifying number (UIN)

Not applicable.

## Guarantor

Wangshu Li.

## Data availability statement

Publicly available datasets were analyzed in this study and all the details are provided in the manuscript. For additional information or queries, interested parties are encouraged to reach out to the corresponding author.

## Provenance and peer review

Not commissioned.

## Supplementary Material

## References

[1] Lamceva J Uljanovs R Strumfa I. The main theories on the pathogenesis of endometriosis. Int J Mol Sci 2023;24:4254. 36901685 10.3390/ijms24054254 PMC10001466

[2] Saunders PTK Horne AW. Endometriosis: etiology, pathobiology, and therapeutic prospects. Cell 2021;184:2807–2824. 34048704 10.1016/j.cell.2021.04.041

[3] Abramiuk M Grywalska E Małkowska P. The role of the immune system in the development of endometriosis. Cells 2022;11:2028. 35805112 10.3390/cells11132028 PMC9265783

[4] Vallvé-Juanico J Houshdaran S Giudice LC. The endometrial immune environment of women with endometriosis. Hum Reprod Update 2019;25:564–591. 31424502 10.1093/humupd/dmz018 PMC6737540

[5] Sanderson E Glymour MM Holmes MV. Mendelian randomization. Nat Rev Methods Primers 2022;2:6. 37325194 10.1038/s43586-021-00092-5 PMC7614635

[6] Wang Y Nicholes K Shih IM. The origin and pathogenesis of endometriosis. Annu Rev Pathol 2020;15:71–95. 31479615 10.1146/annurev-pathmechdis-012419-032654 PMC7980953

[7] Rizner TL. Estrogen metabolism and action in endometriosis. Mol Cell Endocrinol 2009;307:8–18. 19524121 10.1016/j.mce.2009.03.022

[8] Slabe N Meden-Vrtovec H Verdenik I. Cytotoxic T-cells in peripheral blood in women with endometriosis. Geburtshilfe Frauenheilkund 2013;73:1042–1048. 10.1055/s-0033-1350702 PMC3862048 24771894

[9] Schulke L Manconi F Markham R. Endometrial dendritic cell populations during the normal menstrual cycle. Hum Reprod 2008;23:1574–1580. 18285323 10.1093/humrep/den030

[10] Izumi G Koga K Takamura M. Involvement of immune cells in the pathogenesis of endometriosis. J Obstet Gynaecol Res 2018;44:191–198. 29316073 10.1111/jog.13559

