# Supplementary material for: A Mendelian randomization approach to study the causal association between four types of endometriosis and immune cells: experimental studies
Source: Int J Surg. 2024 Jul 3;111(1):1461–5. doi: 10.1097/JS9.0000000000001909 (PMC11745679; doi:10.1097/JS9.0000000000001909)
Supplement: Supplementary file 1 [file js9-111-1461-s001.docx]

**Table 1** The population information of endometriosis involved in this Mendelian randomization.

| Outcome | Ethnic | (case/control) | release | source | SNPs |
| --- | --- | --- | --- | --- | --- |
| ENDOMETRIOSIS_INTESTINE | European | 177/ 68,969 | 2021 | FinnGen R8 | 16,376,157 |
| ENDOMETRIOSIS_OVARY | European | 3,231/68,969 | 2021 | FinnGen R8 | 16,376,686 |
| ENDOMETRIOSIS_PELVICPERITONEUM | European | 2,953/68,969 | 2021 | FinnGen R8 | 16,376,599 |
| ENDOMETRIOSIS_RECTPVAGSEPT_VAGINA | European | 1,360/68,969 | 2021 | FinnGen R8 | 16,376,472 |
