# Supplementary material for: A Mendelian randomization approach to study the causal association between four types of endometriosis and immune cells: experimental studies
Source: Int J Surg. 2024 Jul 3;111(1):1461–5. doi: 10.1097/JS9.0000000000001909 (PMC11745679; doi:10.1097/JS9.0000000000001909)
Supplement: Supplementary file 2 [file js9-111-1461-s002.docx]

**Table S2** Sensitivity analysis of all results has validated the robustness of the observed causal associations**.**

**Type1. Endometriosis of the ovary**

| No. | Exposure | MR-Egger | | IVW | | Horizontal pleiotropy | |
| --- | --- | --- | --- | --- | --- | --- | --- |
|  |  | *Q-val.* | *p-val.* | *Q-val.* | *p-val.* | *Egger_intercept* | *p-val.* |
| 1 | CCR2 on myeloid Dendritic Cell | 0.076 | 0.783 | 2.251 | 0.324 | 0.117 | 0.379 |
| 2 | CCR7 on naive CD4^+^ T cell | 3.99 | 0.136 | 5.83 | 0.12 | 0.079 | 0.438 |
| 3 | CD25 on CD39^+^ CD4^+^ T cell | 3.19 | 0.526 | 3.2 | 0.669 | -0.004 | 0.945 |
| 4 | CD3 on activated & secreting CD4 regulatory T cell | 0.321 | 0.852 | 0.557 | 0.906 | -0.029 | 0.675 |
| 5 | CD3 on activated CD4 regulatory T cell | 0.232 | 0.89 | 0.517 | 0.915 | -0.032 | 0.647 |
| 6 | CD3 on CD39^+^ CD4^+^ T cell | 1.61 | 0.447 | 2.6 | 0.457 | -0.035 | 0.424 |
| 7 | CD3 on CD4 regulatory T cell | 0.271 | 0.873 | 0.461 | 0.927 | -0.025 | 0.705 |
| 8 | CD33^dim^ HLA DR^+^ CD11b^-^ % CD33^dim^ HLA DR^+^ | 0.021 | 0.886 | 1.778 | 0.411 | 0.127 | 0.411 |
| 9 | CD33^dim^ HLA DR^+^ CD11b^+^ %CD33^dim^ HLA DR^+^ | 2.21 | 0.842 | 8.212 | 0.932 | 0.021 | 0.421 |
| 10 | CD39 on CD39^+^ CD4^+^ T cell | 3.9 | 0.272 | 4.07 | 0.397 | -0.009 | 0.747 |
| 11 | CD39 on CD39^+^ secreting CD4 regulatory T cell | 1.86 | 0.602 | 1.97 | 0.742 | 0.008 | 0.766 |
| 12 | CD39^+^activated CD4 regulatory T cell % CD4 regulatory T cell | 3 | 0.7 | 3.15 | 0.789 | 0.009 | 0.712 |
| 13 | CD39^+^ resting CD4 regulatory T cell % resting CD4 regulatory T cell | 5.58 | 0.472 | 6.1 | 0.529 | -0.02 | 0.499 |
| 14 | CD40 on CD14^-^ CD16^+^ monocyte | 4.67 | 0.701 | 4.97 | 0.761 | -0.024 | 0.6 |
| 15 | CD40 on CD14^+^ CD16^-^ monocyte | 7.32 | 0.531 | 9.34 | 0.925 | -0.012 | 0.532 |
| 16 | CD40 on CD14^+^ CD16^+^ monocyte | 6.74 | 0.721 | 5.13 | 0.654 | -0.005 | 0.872 |
| 17 | CD45RA^+^ CD8^+^ T cell Absolute Count | 0.164 | 0.685 | 0.782 | 0.676 | 0.233 | 0.576 |
| 18 | CX3CR1 on CD14^+^ CD16^-^ monocyte | 0.013 | 0.91 | 0.071 | 0.965 | -0.022 | 0.849 |
| 19 | CX3CR1 on CD14^+^ CD16^+^ monocyte | 6.21 | 0.647 | 7.21 | 0.583 | -0.042 | 0.749 |
| 20 | CX3CR1 on monocyte | 7.42 | 0.991 | 0.03 | 0.985 | -0.013 | 0.89 |
| 21 | HLA DR on CD14^-^ CD16^+^ monocyte | 3.27 | 0.774 | 3.61 | 0.824 | 0.02 | 0.582 |
| 22 | HLA DR on CD33dim HLA DR^+^ CD11b^+^ | 0.333 | 0.846 | 1.001 | 0.801 | -0.1 | 0.5 |
| 23 | CCR2 on myeloid Dendritic Cell | 0.076 | 0.783 | 2.251 | 0.324 | 0.117 | 0.379 |

**Type 2. Peritoneal endometriosis**

| No | Exposure | MR-Egger | | IVW | | Horizontal pleiotropy | |
| --- | --- | --- | --- | --- | --- | --- | --- |
|  |  | *Q-val.* | *p-val.* | *Q-val.* | *p-val.* | *Egger_intercept* | *p-val.* |
| 1 | CD25 on B cell | 0.011 | 0.915 | 0.393 | 0.822 | 0.036 | 0.648 |
| 2 | CD25 on IgD^+^ CD24^-^ B cell | 1.8 | 0.615 | 1.8 | 0.0772 | 0.002 | 0.97 |
| 3 | CD25 on IgD^+^ CD38^-^ naive B cell | 0.774 | 0.379 | 0.794 | 0.672 | 0.011 | 0.911 |
| 4 | CD25^++^ CD45RA^+^ CD4 not regulatory T cell Absolute Count | 1.10 | 0.294 | 1.39 | 0.498 | 0.018 | 0.697 |
| 5 | CD28 on CD39^+^ activated CD4 regulatory T cell | 0.049 | 0.825 | 0.856 | 0.652 | 0.083 | 0.534 |
| 6 | CD33 on CD66b^++^ myeloid cell | 3.57 | 0.312 | 3.62 | 0.461 | 0.006 | 0.861 |
| 7 | CD33^dim^ HLA DR^+^ CD11b^-^ %CD33^dim^ HLA DR^+^ | 1.42 | 0.234 | 1.44 | 0.486 | 0.017 | 0.912 |
| 8 | CD33dim HLA DR^+^ CD11b^+^ %CD33dim HLA DR+ | 1.12 | 0.431 | 7.4 | 0.73 | 0.004 | 0.421 |
| 9 | CD40 on CD14^-^ CD16^+^ monocyte | 7.3 | 0.398 | 10.6 | 0.224 | 0.081 | 0.118 |
| 10 | CD40 on CD14^+^ CD16^-^ monocyte | 1.9 | 0.912 | 9.1 | 0.63 | -0.008 | 0.343 |
| 11 | CD40 on CD14^+^ CD16^+^ monocyte | 1.992 | 0.689 | 3.5 | 0.5 | -0.041 | 0.743 |
| 12 | CX3CR1 on CD14^+^ CD16^-^ monocyte | 0.042 | 0.838 | 0.468 | 0.791 | -0.061 | 0.632 |
| 13 | CX3CR1 on CD14^+^ CD16^+^ monocyte | 4.2 | 0.765 | 8.32 | 0.942 | 0.032 | 0.621 |
| 14 | CX3CR1 on monocyte | 0.001 | 0.970 | 0.345 | 0.842 | -0.047 | 0.663 |
| 15 | HLA DR on HLA DR^+^ Natural Killer | 3.48 | 0.481 | 3.66 | 0.600 | -0.022 | 0.698 |
| 16 | Natural Killer T Absolute Count | 0.751 | 0.861 | 0.762 | 0.943 | 0.004 | 0.921 |
| 17 | SSC-A on CD14^+^ monocyte | 3.99 | 0.407 | 5.09 | 0.405 | -0.046 | 0.353 |
| 18 | CD25 on B cell | 0.011 | 0.915 | 0.393 | 0.822 | 0.036 | 0.648 |

**Type. 3. Other types (intestinal endometriosis)**

| No | Exposure | MR-Egger | | IVW | | Horizontal pleiotropy | |
| --- | --- | --- | --- | --- | --- | --- | --- |
|  |  | *Q-val.* | *p-val.* | *Q-val.* | *p-val.* | *Egger_intercept* | *p-val.* |
| 1 | CD11c on myeloid Dendritic Cell | 2.99 | 0.393 | 2.99 | 0.560 | 0.005 | 0.984 |
| 2 | CD127 on CD28^+^ CD4^+^ T cell | 0.367 | 0.545 | 0.402 | 0.818 | -0.076 | 0.882 |
| 3 | CD127 on CD45RA^+^ CD4^+^ T cell | 2.07 | 0.355 | 2.67 | 0.446 | -0.391 | 0.527 |
| 4 | CD16 on CD14^+^ CD16^+^ monocyte | 1.29 | 0.732 | 1.93 | 0.748 | 0.208 | 0.481 |
| 5 | CD25 on IgD^+^ CD38^-^ B cell | 0.223 | 0.637 | 1.546 | 0.462 | -0.258 | 0.456 |
| 6 | CD33^dim^ HLA DR^+^ CD11b^-^ %CD33^dim^ HLA DR^+^ | 0.745 | 0.388 | 1.022 | 0.600 | 0.206 | 0.692 |
| 7 | CD33^dim^ HLA DR^+^ CD11b^+^ %CD33^dim^ HLA DR^+^ | 0.82 | 0.41 | 1.332 | 0.652 | 0.433 | 0.652 |
| 8 | CD45RA^+^ CD8^+^ T cell Absolute Count | 1.53 | 0.216 | 1.68 | 0.431 | 0.472 | 0.804 |
| 9 | CX3CR1 on CD14^+^ CD16^+^ monocyte | 1.13 | 0.288 | 1.26 | 0.534 | -0.066 | 0.792 |
| 10 | CX3CR1 on monocyte | 0.464 | 0.496 | 1.374 | 0.503 | -0.296 | 0.515 |
| 11 | HLA DR^+^ Natural Killer %Natural Killer | 3.94 | 0.268 | 4.90 | 0.298 | 0.1 | 0.457 |
| 12 | HLA DR^+^ Natural Killer Absolute Count | 2.24 | 0.327 | 2.52 | 0.471 | 0.113 | 0.663 |
| 13 | CD11c on myeloid Dendritic Cell | 2.99 | 0.393 | 2.99 | 0.560 | 0.005 | 0.984 |

**Type 4. Deep infiltrating endometriosis**

| No | Exposure | MR-Egger | | IVW | | Horizontal pleiotropy | |
| --- | --- | --- | --- | --- | --- | --- | --- |
|  |  | *Q-val.* | *p-val.* | *Q-val.* | *p-val.* | *Egger_intercept* | *p-val.* |
| 1 | CD25^++^ CD8^+^ T cell Absolute Count | 1.09 | 0.297 | 1.7 | 0.427 | -0.049 | 0.589 |
| 2 | CD3 on Effector Memory CD8^+^ T cell | 0.509 | 0.476 | 2.021 | 0.364 | -0.148 | 0.435 |
