# Supplementary material for: A Mendelian randomization approach to study the causal association between four types of endometriosis and immune cells: experimental studies
Source: Int J Surg. 2024 Jul 3;111(1):1461–5. doi: 10.1097/JS9.0000000000001909 (PMC11745679; doi:10.1097/JS9.0000000000001909)
Supplement: Supplementary file 3 [file js9-111-1461-s003.docx]

**Supplementary Table S3.**

**Type 1**. Endometriosis of the ovary

| Exposure | Panel | b | pval | or | or_lci95 | or_uci95 | mr-egger-Q | mr-egger-Qval. | Ivw-Q | Ivw-Qval. | Egger-intercept | Pval. |
| --- | --- | --- | --- | --- | --- | --- | --- | --- | --- | --- | --- | --- |
| CCR2 on myeloid Dendritic Cell | cDC | 0.132 | 0.041 | 1.141 | 1.005 | 1.296 | 0.076 | 0.783 | 2.251 | 0.324 | 0.117 | 0.379 |
| CCR7 on naive CD4^+^ T cell | Maturation stages of T cell | -0.229 | 0.009 | 0.796 | 0.671 | 0.943 | 3.990 | 0.136 | 5.830 | 0.120 | 0.079 | 0.438 |
| CD25 on CD39^+^ CD4^+^ T cell | Treg | -0.083 | 0.017 | 0.921 | 0.860 | 0.985 | 3.190 | 0.526 | 3.200 | 0.669 | -0.004 | 0.945 |
| CD3 on activated & secreting CD4 regulatory T cell | Treg | 0.086 | 0.032 | 1.089 | 1.007 | 1.178 | 0.321 | 0.852 | 0.557 | 0.906 | -0.029 | 0.675 |
| CD3 on activated CD4 regulatory T cell | Treg | 0.087 | 0.031 | 1.091 | 1.008 | 1.181 | 0.232 | 0.890 | 0.517 | 0.915 | -0.032 | 0.647 |
| CD3 on CD39^+^ CD4^+^ T cell | Treg | 0.081 | 0.043 | 1.084 | 1.003 | 1.172 | 1.610 | 0.447 | 2.600 | 0.457 | -0.035 | 0.424 |
| CD3 on CD4 regulatory T cell | Treg | 0.087 | 0.030 | 1.091 | 1.009 | 1.180 | 0.271 | 0.873 | 0.461 | 0.927 | -0.025 | 0.705 |
| CD33^dim^ HLA DR^+^ CD11b^-^ %CD33^dim^ HLA DR^+^ | Myeloid cell | 0.124 | 0.001 | 1.132 | 1.050 | 1.221 | 0.021 | 0.886 | 1.778 | 0.411 | 0.127 | 0.411 |
| CD33^dim^ HLA DR^+^ CD11b^+^ %CD33^dim^ HLA DR^+^ | Myeloid cell | -0.124 | 0.001 | 0.883 | 0.819 | 0.952 |  |  |  |  |  |  |
| CD39 on CD39^+^ CD4^+^ T cell | Treg | 0.056 | 0.018 | 1.058 | 1.010 | 1.108 | 3.900 | 0.272 | 4.070 | 0.397 | -0.009 | 0.747 |
| CD39 on CD39^+^ secreting CD4 regulatory T cell | Treg | 0.060 | 0.009 | 1.062 | 1.015 | 1.111 | 1.860 | 0.602 | 1.970 | 0.742 | 0.008 | 0.766 |
| CD39^+^ activated CD4 regulatory T cell %CD4 regulatory T cell | Treg | 0.061 | 0.013 | 1.063 | 1.013 | 1.116 | 3.000 | 0.700 | 3.150 | 0.789 | 0.009 | 0.712 |
| CD39^+^ resting CD4 regulatory T cell %resting CD4 regulatory T cell | Treg | 0.060 | 0.014 | 1.062 | 1.012 | 1.114 | 5.580 | 0.472 | 6.100 | 0.529 | -0.020 | 0.499 |
| CD40 on CD14^-^ CD16^+^ monocyte | Treg | -0.083 | 0.005 | 0.920 | 0.869 | 0.975 | 4.670 | 0.701 | 4.970 | 0.761 | -0.024 | 0.600 |
| CD40 on CD14^+^ CD16^-^ monocyte | Monocyte | -0.096 | 0.006 | 0.909 | 0.848 | 0.973 |  |  |  |  |  |  |
| CD40 on CD14^+^ CD16^+^ monocyte | Monocyte | -0.073 | 0.024 | 0.929 | 0.872 | 0.990 |  |  |  |  |  |  |
| CD45RA^+^ CD8^+^ T cell Absolute Count | Monocyte | -0.294 | 0.014 | 0.745 | 0.590 | 0.941 | 0.164 | 0.685 | 0.782 | 0.676 | 0.233 | 0.576 |
| CX3CR1 on CD14^+^ CD16^-^ monocyte | Maturation stages of T cell | 0.103 | 0.027 | 1.109 | 1.012 | 1.216 | 0.013 | 0.910 | 0.071 | 0.965 | -0.022 | 0.849 |
| CX3CR1 on CD14^+^ CD16^+^ monocyte | Monocyte | 0.112 | 0.028 | 1.119 | 1.012 | 1.237 |  |  |  |  |  |  |
| CX3CR1 on monocyte | Monocyte | 0.114 | 0.027 | 1.121 | 1.013 | 1.241 | 0.000 | 0.991 | 0.030 | 0.985 | -0.013 | 0.890 |
| HLA DR on CD14^-^ CD16^+^ monocyte | Monocyte | -0.102 | 0.004 | 0.903 | 0.841 | 0.969 | 3.270 | 0.774 | 3.610 | 0.824 | 0.020 | 0.582 |
| HLA DR on CD33^dim^ HLA DR^+^ CD11b^+^ | Monocyte | -0.126 | 0.003 | 0.882 | 0.813 | 0.957 | 0.333 | 0.846 | 1.001 | 0.801 | -0.100 | 0.500 |

**Type 2.** Peritoneal Endometriosis

| Exposure | Panel | b | se | pval | lo_ci | up_ci | or | or_lci95 | or_uci95 | mr-egger-Q | mr-egger-Qvalue | Ivw-Q | Ivw-Qval. | Egger-intercept | Pval. |
| --- | --- | --- | --- | --- | --- | --- | --- | --- | --- | --- | --- | --- | --- | --- | --- |
| CD25 on B cell | B cell | 0.219 | 0.083 | 0.008 | 0.057 | 0.381 | 1.245 | 1.059 | 1.464 | 0.011 | 0.915 | 0.393 | 0.822 | 0.036 | 0.648 |
| CD25 on IgD^+^ CD24^-^ B cell | B cell | 0.173 | 0.067 | 0.010 | 0.042 | 0.305 | 1.189 | 1.043 | 1.356 | 1.800 | 0.615 | 1.800 | 0.077 | 0.002 | 0.970 |
| CD25 on IgD^+^ CD38^-^ naive B cell | B cell | 0.167 | 0.080 | 0.036 | 0.011 | 0.323 | 1.182 | 1.011 | 1.381 | 0.774 | 0.379 | 0.794 | 0.672 | 0.011 | 0.911 |
| CD25^++^ CD45RA^+^ CD4 not regulatory T cell Absolute Count | Treg | -0.154 | 0.068 | 0.024 | -0.288 | -0.020 | 0.857 | 0.750 | 0.980 | 1.100 | 0.294 | 1.390 | 0.498 | 0.018 | 0.697 |
| CD28 on CD39^+^ activated CD4 regulatory T cell | Treg | 0.150 | 0.076 | 0.048 | 0.001 | 0.300 | 1.162 | 1.001 | 1.349 | 0.049 | 0.825 | 0.856 | 0.652 | 0.083 | 0.534 |
| CD33 on CD66b^++^ myeloid cell | Myeloid | -0.053 | 0.027 | 0.048 | -0.105 | 0.000 | 0.949 | 0.900 | 1.000 | 3.570 | 0.312 | 3.620 | 0.461 | 0.006 | 0.861 |
| CD33^dim^ HLA DR^+^ CD11b^-^ %CD33^dim^ HLA DR^+^ | Myeloid | 0.126 | 0.040 | 0.002 | 0.047 | 0.204 | 1.134 | 1.048 | 1.227 | 1.420 | 0.234 | 1.440 | 0.486 | 0.017 | 0.912 |
| CD33^dim^ HLA DR^+^ CD11b^+^ %CD33^dim^ HLA DR^+^ | Myeloid | -0.125 | 0.040 | 0.002 | -0.204 | -0.047 | 0.882 | 0.816 | 0.954 |  |  |  |  |  |  |
| CD40 on CD14^-^ CD16^+^ monocyte | Monocyte | -0.075 | 0.035 | 0.035 | -0.144 | -0.005 | 0.928 | 0.866 | 0.995 | 7.300 | 0.398 | 10.600 | 0.224 | 0.081 | 0.118 |
| CD40 on CD14^+^ CD16^-^ monocyte | Monocyte | -0.093 | 0.041 | 0.024 | -0.174 | -0.012 | 0.911 | 0.840 | 0.988 |  |  |  |  |  |  |
| CD40 on CD14^+^ CD16^+^ monocyte | Monocyte | -0.090 | 0.034 | 0.008 | -0.156 | -0.023 | 0.914 | 0.855 | 0.977 |  |  |  |  |  |  |
| CX3CR1 on CD14^+^ CD16^-^ monocyte | Monocyte | 0.119 | 0.049 | 0.015 | 0.023 | 0.215 | 1.126 | 1.023 | 1.240 | 0.042 | 0.838 | 0.468 | 0.791 | -0.061 | 0.632 |
| CX3CR1 on CD14^+^ CD16^+^ monocyte | Monocyte | 0.133 | 0.053 | 0.013 | 0.028 | 0.238 | 1.143 | 1.029 | 1.269 |  |  |  |  |  |  |
| CX3CR1 on monocyte | Monocyte | 0.132 | 0.054 | 0.014 | 0.027 | 0.238 | 1.141 | 1.027 | 1.269 | 0.001 | 0.970 | 0.345 | 0.842 | -0.047 | 0.663 |
| HLA DR on HLA DR^+^ Natural Killer | TBNK | -0.118 | 0.051 | 0.020 | -0.217 | -0.019 | 0.889 | 0.805 | 0.981 | 3.480 | 0.481 | 3.660 | 0.600 | -0.022 | 0.698 |
| Natural Killer T Absolute Count | TBNK | 0.258 | 0.082 | 0.002 | 0.097 | 0.418 | 1.294 | 1.102 | 1.519 | 0.751 | 0.861 | 0.762 | 0.943 | 0.004 | 0.921 |
| SSC-A on CD14^+^ monocyte | TBNK | -0.096 | 0.047 | 0.042 | -0.188 | -0.004 | 0.909 | 0.829 | 0.997 | 3.990 | 0.407 | 5.090 | 0.405 | -0.046 | 0.353 |

**Type 3.** Other Types (Intestinal)

| Exposure | Panel | b | Pval. | or | or_lci95 | or_uci95 | mr-egger-Q | mr-egger-Qval. | Ivw-Q | Ivw-Qval. | Egger-intercept | Pval. |
| --- | --- | --- | --- | --- | --- | --- | --- | --- | --- | --- | --- | --- |
| CD11c on myeloid Dendritic Cell | cDC | -0.687 | 0.023 | 0.503 | 0.278 | 0.910 | 2.990 | 0.393 | 2.990 | 0.560 | 0.005 | 0.984 |
| CD127 on CD28^+^ CD4^+^ T cell | Treg | -0.870 | 0.019 | 0.419 | 0.203 | 0.865 | 0.367 | 0.545 | 0.402 | 0.818 | -0.076 | 0.882 |
| CD127 on CD45RA^+^ CD4^+^ T cell | Treg | -0.639 | 0.018 | 0.528 | 0.311 | 0.895 | 2.070 | 0.355 | 2.670 | 0.446 | -0.391 | 0.527 |
| CD16 on CD14^+^ CD16^+^ monocyte | Monocyte | 0.375 | 0.040 | 1.455 | 1.018 | 2.081 | 1.290 | 0.732 | 1.930 | 0.748 | 0.208 | 0.481 |
| CD25 on IgD^+^ CD38^-^ B cell | B cell | 0.854 | 0.038 | 2.348 | 1.048 | 5.262 | 0.223 | 0.637 | 1.546 | 0.462 | -0.258 | 0.456 |
| CD33^dim^ HLA DR^+^ CD11b^-^ %CD33^dim^ HLA DR^+^ | Myeloid cell | 0.345 | 0.027 | 1.412 | 1.040 | 1.917 | 0.745 | 0.388 | 1.022 | 0.600 | 0.206 | 0.692 |
| CD33^dim^ HLA DR^+^ CD11b^+^ %CD33^dim^ HLA DR^+^ | Myeloid cell | -0.344 | 0.027 | 0.709 | 0.523 | 0.961 | 0.820 | 0.410 | 1.332 | 0.652 | 0.433 | 0.652 |
| CD45RA^+^ CD8^+^ T cell Absolute Count | Maturation stages of T cell | -1.068 | 0.026 | 0.344 | 0.134 | 0.882 | 1.530 | 0.216 | 1.680 | 0.431 | 0.472 | 0.804 |
| CX3CR1 on CD14^+^ CD16^+^ monocyte | Monocyte | 0.423 | 0.042 | 1.527 | 1.016 | 2.295 | 1.130 | 0.288 | 1.260 | 0.534 | -0.066 | 0.792 |
| CX3CR1 on monocyte | Monocyte | 0.421 | 0.045 | 1.523 | 1.010 | 2.296 | 0.464 | 0.496 | 1.374 | 0.503 | -0.296 | 0.515 |
| HLA DR^+^ Natural Killer %Natural Killer | TBNK | 0.480 | 0.027 | 1.616 | 1.056 | 2.475 | 3.940 | 0.268 | 4.900 | 0.298 | 0.100 | 0.457 |
| HLA DR^+^ Natural Killer Absolute Count | TBNK | 0.426 | 0.035 | 1.531 | 1.031 | 2.273 | 2.240 | 0.327 | 2.520 | 0.471 | 0.113 | 0.663 |

**Type 4.** Deep Infiltrating Endometriosis

| Exposure | Panel | b | se | Pval. | or | or_lci95 | or_uci95 | mr-egger-Q | mr-egger-Qval. | Ivw-Q | Ivw-Qval. | Egger-intercept | Pval. |
| --- | --- | --- | --- | --- | --- | --- | --- | --- | --- | --- | --- | --- | --- |
| CD25^++^ CD8^+^ T cell Absolute Count | Treg | 0.219 | 0.107 | 0.042 | 1.245 | 1.008 | 1.536 | 1.090 | 0.297 | 1.700 | 0.427 | -0.049 | 0.589 |
| CD3 on Effector Memory CD8^+^ T cell | Maturation stages of T cell | 0.206 | 0.104 | 0.048 | 1.228 | 1.002 | 1.506 | 0.509 | 0.476 | 2.021 | 0.364 | -0.148 | 0.435 |
